# A comparative cross-sectional study of the impact of COVID-19 pandemic on obstetrics and gynecology admissions in Croatia

**DOI:** 10.3389/fmed.2025.1505387

**Published:** 2025-02-14

**Authors:** Karolina Kalanj, Mislav Mikuš, Mirta Peček, Ante Orbanić, Rick Marshall, Stjepan Orešković, Slavko Orešković

**Affiliations:** ^1^Andrija Štampar School of Public Health, School of Medicine, University of Zagreb, Zagreb, Croatia; ^2^Department of Obstetrics and Gynecology, University Hospital Centre, Zagreb, Croatia; ^3^Institute for Emergency Medicine Virovitica- Podravina County, Virovitica, Croatia; ^4^Independent Consultant, Zagreb, Croatia; ^5^Independent Consultant in Health System Funding Models, Eaglehawk Neck, TAS, Australia; ^6^School of Medicine, University of Zagreb, Zagreb, Croatia

**Keywords:** AR-DRG, COVID-19, data transparency, gynecology, health system response, inpatient care, obstetrics, pandemic

## Abstract

**Background:**

The COVID-19 pandemic placed unprecedented pressure on healthcare systems worldwide and altered patients' perceptions of the system's ability to protect them from virus transmission. One significant consequence was a marked decline in hospital activity, a trend observed globally. This study aims to evaluate the impact of COVID-19 on hospitalization rates among patients with gynecological disorders in Croatia. It compares the number of patients treated surgically vs. conservatively before the pandemic (2017–2019) and during the pandemic (2020–2022) using the Diagnostic-Related Group (DRG) patient classification system. The DRG system is designed to group patients based on similar clinical conditions, complexity, and resource utilization. Hospital activity categorized by DRGs was analyzed to assess the impact of the COVID-19 pandemic on case volumes within DRG groups associated with gynecological and obstetric disorders.

**Materials and methods:**

We conducted a comparative descriptive cross sectional study of the pre-post type according to STROBE guidelines to determine the impact of COVID-19 pandemic on hospital admission rates for patients with conditions associated with illnesses and abnormalities of the female reproductive system, as well as pregnancy, delivery, and the puerperium. The publicly available data collected by Croatian Institute of Public Health (CIPH) and the Croatian Health Insurance Fund (CHIF) were the main data source for this study. All gynecological hospital admissions in Croatia were grouped based on the Australian Refined Diagnosis Related Groups (AR-DRGs) and analyzed over two time periods: before (2017–2019) and during the pandemic (2020–2022).

**Results:**

The average number of gynecological patients in all hospitals during the pandemic was 62,257 compared to pre-pandemic when the average number of patients was 71,519, a decrease of 15.5%. The results show a 10.56% decrease in the total number of non-surgical admissions and 12.8% decrease of surgical admissions across the hospital network during the pandemic (2020–2022).

**Conclusion:**

The COVID-19 pandemic led to a significant decline in inpatient treatments in gynecology and obstetrics departments in Croatia. Our findings highlight the need for obstetrics and gynecology practitioners to develop innovative strategies to maintain or enhance patient access to appropriate care while ensuring stringent infection prevention measures for both patients and healthcare personnel. Furthermore, investing in healthcare system resilience is crucial to maintaining core functions during future crises. The lessons learned from the COVID-19 pandemic provide a valuable opportunity to fortify healthcare systems and must not be ignored.

## Introduction

The COVID-19 outbreak, that originated in China, rapidly spread worldwide in 2020, culminating in a global pandemic. In response, governments urgently worked to reorganize their healthcare resources to meet the escalating demand for the treatment and management of COVID-19 patients (1).

Simultaneously, a multidisciplinary efforts and research by experts from various medical specialties led to the publication of academic papers that examined the direct impact of COVID-19 on specific illness categories and in the process proposed the postponement of non-urgent medical treatments and surgical procedures (2, 3). This recommendation was intended among other things, to alleviate the overcrowding in hospitals and healthcare facilities, and thereby the likely spread of the infection. The result of all these control activities was that governments introduced extensive public health measures which included social isolation, border closures, school closures, procedures to isolate symptomatic persons and their social contacts, and population lockdowns save for necessary internal transit (4). The duration of the lockdowns, depended on the spread of the SARS-CoV-2 pandemic in jurisdictions.

One consequence of these measures was that patients reduced or stopped seeking care and visiting medical care facilities and hospitals to minimize their risk of virus exposure. This avoidance was particularly pronounced among older age groups, who experienced heightened anxiety about COVID-19 due to their increased risk of severe outcomes. This demographic was especially proactive in adopting preventive measures, such as avoiding crowded areas, wearing protective masks, and practicing frequent handwashing (5).

As the decline in hospital activity became evident, innovative approaches were implemented to ensure continued access to healthcare services. Contactless solutions, such as telemedicine, were introduced to bridge the gap and maintain patient care (6). Telemedicine, as defined by the World Health Organization, is the use of information and communication technology to offer medical treatments where distance is a significant problem for all medical professionals (7).

Before COVID-19, the use of telemedicine in gynecology was primarily limited to activities related to prenatal care or as part of sexually transmitted disease awareness campaigns, often delivered through online learning courses or webinars. As a result, clinicians specializing in gynecology and obstetrics had minimal experience with telemedicine, with their lack of knowledge and expertise presenting a significant barrier to its broader adoption. Additionally, other challenges in implementing telemedicine within gynecology included high administrative costs and inadequate reimbursement structures, further hindering its integration into routine practice.

Therefore, the COVID-19 pandemic highlighted, among other issues, the underrepresentation of telemedicine in gynecology compared to its more established use in fields like internal medicine, anesthesia, and intensive care (8, 9). More studies into the viewpoints and preferences of doctors, patients, and other telemedicine users in gynecology are critical in the field of telemedicine. This can serve as the foundation for the specific patient and physician-tailored telemedicine solutions, as well as the development and improvement of patient triage processes for digital or analog consultation hours (8, 10, 11).

As the pandemic unfolded it became clear that COVID-19 had an adverse impact on maternal-fetal wellbeing and obstetric treatment (12), and pregnancy is being considered a risk factor for a severe course of COVID-19 (13). Furthermore, COVID-19 infection during pregnancy has been associated with changes in pregnancy management (14), an increase in miscarriage pregnancy outcomes (15, 16), and need for hospitalization (17) and preterm delivery in more than half of affected cases (18). As pregnancy is associated with a higher risk of severe COVID-19 compared with the non-pregnant population, including pneumonia, admission to the ICU and death, a thorough follow-up of pregnant women with SARS-CoV-2 infection is needed in order to identify those cases at higher risk of developing the most severe spectrum of disease (19).

Croatia's strategy for COVID-19 was generally similar to those of other European countries. According to the Government Stringency Index (GSI), Croatia's mitigation measures were initially quite stringent (with a GSI close to 100), but by late November 2020, they could be considered to be relatively mild (with a GSI around 50 and later on 30).

This study aims to evaluate the direct impact of COVID-19 on the number of hospitalized patients with gynecological illnesses contained in Chapter 2 (Neoplasms), Chapter 14 (parts related to Diseases of the female genitourinary system) and Chapter 15 (Pregnancy, Childbirth and Puerperium) of International Classification of Diseases, Tenth Revision at the secondary, and tertiary healthcare levels in Croatia, in the period before (2017–2019) and during the pandemic (2020–2022). By determining which types of patients were mostly affected may serve as a guiding principle in the process of enhancing resilience of health care system delivery.

## Materials and methods

### Study design and setting

This study is comparative, descriptive cross-sectional study of the pre-post type according to STROBE guidelines.

The data were acquired from databases maintained by the Croatian Institute of Public Health (CIPH) and the Croatian Health Insurance Fund (CHIF), both of which are open to the public (20). Croatian patient classification system is based on the Australian Refined–Diagnosis Related Group version 5.2 (AR-DRG). AR-DRGs is a classification that provides a clinically meaningful way to group admitted patients with similar diagnosis and similar resource consumption into the same group (21). The AR-DRG structure is based on 23 Major Diagnostic Categories (MDC) defined by the principal diagnoses, which is the primary reason for patient being admitted in hospital.

Each of the MDC representing a different body system or etiology is defined by the principle diagnosis which represent the main reason for patient being admitted in the hospital. In this study, the main interest was the MDC13^1^ and MDC14^2^ groups because they include diseases and disorders of the female reproductive system, as well as pregnancy, childbirth, and the puerperium, respectively (22).

### Participant eligibility criteria

The Australian Refined Diagnosis-Related Groups (AR-DRG) system has been used in Croatia since 2009 as a patient classification system primarily aimed at reporting inpatient activity within the acute hospital network for reimbursement purposes. A key advantage of employing a well-structured classification system like AR-DRG is the comprehensiveness of the data collected. Each episode of care for an admitted patient is coded according to specific standards, ensuring that the principal diagnosis, additional diagnoses, and both operative and non-operative procedures, such as endoscopies, are accurately recorded.

In this system, the principal and additional diagnosis are coded using the International Classification of Diseases, Tenth Revision, Australian modification (ICD 10–AM) Tabular List of diseases that contains the disease classification itself at the three, four and five character levels. Procedures are coded using the Australian Classifications of interventions (ACHI) and the structure of procedure classifications is based on anatomy rather than surgical specialty. Every admitted patient is included in the Croatian DRG data base, and therefore only the inpatient case is included in the database used in the study.

Since the purpose of our study is to examine the impact of pandemic on inpatient admissions reported by acute hospital network, we selected the timeline for data analysis as the period 2017–2022 inclusive. These hospitals serve a population of 3.9 million people providing for 96% of all inpatient activity. The study included 24 secondary-level hospitals and nine tertiary-level hospitals and all facilities included are publicly owned. These hospitals represent almost all gynecological and maternity inpatient care.

Each episode of care related to diseases and disorders of the female reproductive system was grouped into its proper AR-DRG group based on the main reason for the patient's admission, and as a consequence, changes in acute patient admission before and during the COVID-19 pandemic were noticed. Because the data utilized in this study were anonymized and made available as public information from CHIF and the CIPH, we did not require informed consent or ethical approval.

### Data and statistical analysis

The average number of inpatient cases was calculated for 3 years (2017–2019) before the pandemic and 3 years (2020–2022) during the period of the pandemic. We used DRG data grouped in MDC 13 and MDC 14 in order to determine the extent to which, and for which conditions the onset of pandemic altered the pre COVID-19 hospital activity across the hospital network. The hospital admission incidence rates were calculated by dividing the average number of cases during each period (2017–2019 for pre-pandemic years and 2020–2022 for pandemic years) by the average total population of Croatia during the respective periods. The reason why we used the average total population as denominator is related to the fact that DRG data base includes 96% of the country's inpatent activity and this was consistent during the study timeframe periods. The consistency in female population data across both time periods (51.8%) based on the Croatian Bureau of Statistics ensures comparability of the calculated incidence rates.

The hospital admission incidence rate was then determined by dividing the average number of cases during a certain period by the average total population (2017–2019 and 2020–2022). To compare the incidence rates of events (hospital admissions) occurring in pre-pandemic and pandemic period, the incidence rate ratio (IRR) was used as a relative difference measure. The incidence rate ratio (IRR) was calculated as a ratio of the incidence rate for 2020–2022 to that for 2017–2019 for each analyzed DRGs that belongs to MDC 13 and 14. Using the 2-by-2 Chi-square test, the incidence rate between the two time periods was compared.

The Wald technique was used to construct the 95% confidence intervals based on an investigation of whether the IRR was equal to one (i.e., the admission incidence rate in 2020–2022 being equal to that in 2017–2019). Microsoft Excel was used to calculate average values and rate change while every statistical analysis was carried out using R (R Core Team, Austria) (23). No adjustments for seasonal effects and autocorellation were needed as data were compared by calendar year (6 years in total) and therefore a constant variance was assumed. A *p*-value of 0.05 or less was considered statistically significant (two-tailed).

## Results

The average number of gynecological patients in all hospitals during the pandemic (2020–2022) was 62,257 of which 34,987 (56.2%) were treated at the tertiary healthcare level, compared to pre-pandemic (2017–2019) when the average number of patients in all hospitals was 71,519 of which 40,827 (57.09%) were treated at the tertiary healthcare level. The rate change is −15.5%, similar for both health care levels (−17.5%, −12.5%, *p* < 0.0001, respectively).

Among all patients during the pandemic years, 27,540 (44.24%) of them were treated surgically. 15,773 (57.27%) were treated surgically at the tertiary healthcare level. Compared to pre-pandemic years, 31,578 (44.15%) patients were treated surgically. Among them, 18,793 (59.51%) were treated at the tertiary healthcare level.

During the pandemic, there were 14,110 patients treated surgically because of conditions related to pregnancy, childbirth, and puerperium. Compared to the pre-pandemic period, there is an average drop of 6.64%, when the total number of patients was 15,113. During the pandemic, 7,887 (55.9%) patients were surgically treated at the tertiary healthcare level, and 6,223 (44.1%) at the secondary. The number of patients dropped by 19% at the tertiary and by 3% at the secondary health care level.

The decrease greater than average is related to all subcategories in this group, except O03Z in which a decrease of 1% was observed (*p* = 0.953463), and O01B and O01C where an increase of 3% and 10% were observed (*p* = 0.443471, *p* < 0.0001, respectively).

Table 1 compares the average number of total surgical admissions during the pre-pandemic (2017–2019) and pandemic years (2020–2022) related to pregnancy, childbirth, and the puerperium.

**Table 1:** Comparison of surgical DRGs related to pregnancy, childbirth, and the puerperium done during pre-pandemic (2017–2019) and pandemic (2020–2022).

**Codes**	**2017–2019**		**2020–2022**		**2017–2019**	**2020–2022**	**Pre-pandemic–pandemic comparison**			***p*-value**
	**T**	**S**	**T**	**S**	**Average all hospitals**	**Average all hospitals**	**% Rate change T**	**% Rate change S**	**% Rate change all**	
O01A	118	23	84	28	141	112	−28%	20%	−20%	0.071318148
O01B	1,136	617	1,081	718	1,753	1,798	−5%	16%	3%	0.443471419
O01C	3,955	2,793	4,425	2,986	6,747	7,411	12%	7%	10%	2.43921E−08
O02A	143	63	90	52	206	141	−37%	−18%	−32%	0.000490236
O02B	2,169	2,025	1,445	1,683	4,194	3,127	−33%	−17%	−25%	1.14159E−35
O03Z	145	117	137	123	262	260	−6%	6%	−1%	0.953463139
O04Z	174	136	130	127	311	257	−25%	−7%	−17%	0.025232994
O05Z	722	778	496	506	1,500	1,002	−31%	−35%	−33%	2.5214E−23

Codes: O01A–O05Z refers to surgical DRGs related to pregnancy, child birth and puerperium; A cases with catastrophic complications and comorbidities, B cases without catastrophic or severe complications and comorbidities, Z-no comorbidities or complications taken into account, procedural DRG group.

T, tertiary hospitals (University Clinical Centers, Clinical hospitals); S, secondary hospitals (General and County hospitals).

The gynecological patients were treated surgically and conservatively at both healthcare levels. Based on AR-DRG structure, surgical cases are presented with the following ARDRG groups: N01Z-N11B, O01A-O05Z, and patients treated conservatively with groups N60A-N62B, O60A-O66B.

**Figure 1:**
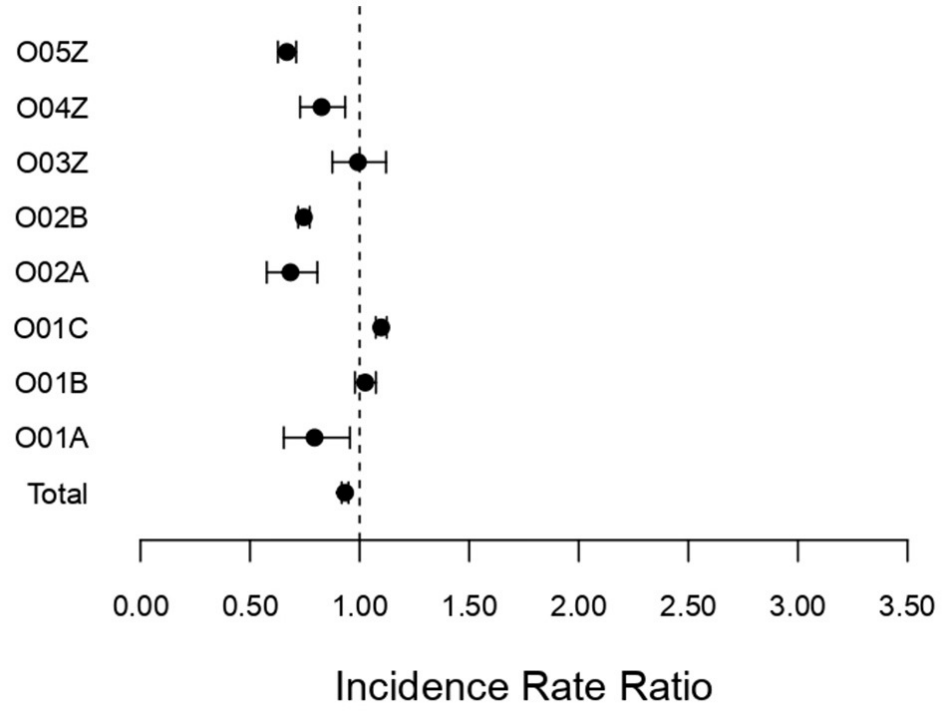
Incidence rate ratio (IRR) for surgical DRGs (O01A–O05Z) associated with pregnancy, childbirth, and the puerperium during the pandemic (2020–2022) compared to pre-pandemic (2017–2019); incidence rate ratio with 95% confidence limits.

Figure 1 shows the corresponding IRRs calculated for surgical DRGs associated with pregnancy, childbirth, and the puerperium.

During the pandemic, there were 32,191 patients treated non-surgically because of conditions related to pregnancy, childbirth, and puerperium. Compared to the pre-pandemic period, there is an average drop of 10.56% when the total number of patients was 35,991. During the pandemic, 17,781 (55.23%) patients were non-surgically treated at the tertiary health care level, and 14,409 (44.76%) at the secondary. The number of patients dropped by 17% at the tertiary and by 11.68% at the secondary health care level.

The decrease greater than average is related to all subcategories in this group, except O66B in which a decrease of 9% was observed (*p* = 0.450941), and O60C and O63Z where an increase of 32% and 52% were observed (*p* = < 0.0001).

Table 2 compares the average number of total non-surgical admissions during the pre-pandemic (2017–2019) and pandemic years (2020–2022) related to pregnancy, childbirth, and puerperium.

**Table 2:** Comparison of non-surgical DRGs related to pregnancy, childbirth, and the puerperium done during pre-pandemic (2017–2019) and pandemic (2020–2022).

**Codes**	**2017–2019**		**2020–2022**		**2017–2019**	**2020–2022**	**Pre-pandemic–pandemic comparison**			***p*-value**
	**T**	**S**	**T**	**S**	**Average all hospitals**	**Average all hospitals**	**% Rate change T**	**% Rate change S**	**% Rate change all**	
O60A	199	145	130	120	344	250	−34%	−18%	−27%	0.00011485
O60B	4,949	5,785	2,430	4,294	10,734	6,724	−51%	−26%	−37%	2.209E−202
O60C	7,306	3,848	9,652	5,078	11,154	14,729	32%	32%	32%	1.9511E−109
O61Z	205	237	152	232	442	384	−26%	−2%	−13%	0.042347807
O63Z	303	326	414	539	629	953	37%	65%	52%	3.71011E−16
O64A	709	538	520	428	1,246	947	−27%	−20%	−24%	1.72637E−10
O64B	191	190	135	179	381	314	−29%	−6%	−18%	0.010665037
O66A	5,759	5,162	4,309	3,453	10,921	7,762	−25%	−33%	−29%	3.9809E−118
O66B	55	85	40	88	140	128	−28%	4%	−9%	0.45094098

Codes: O60A–O66B refers to no surgical DRGs related to pregnancy, child birth and puerperium; A cases with catastrophic complications and comorbidities, B cases without catastrophic or severe complications and comorbidities, Z-no comorbidities or complications taken into account, procedural DRG group.

T, tertiary hospitals (University Clinical Centers, Clinical hospitals); S, secondary hospitals (General and County hospitals).

**Figure 2:**
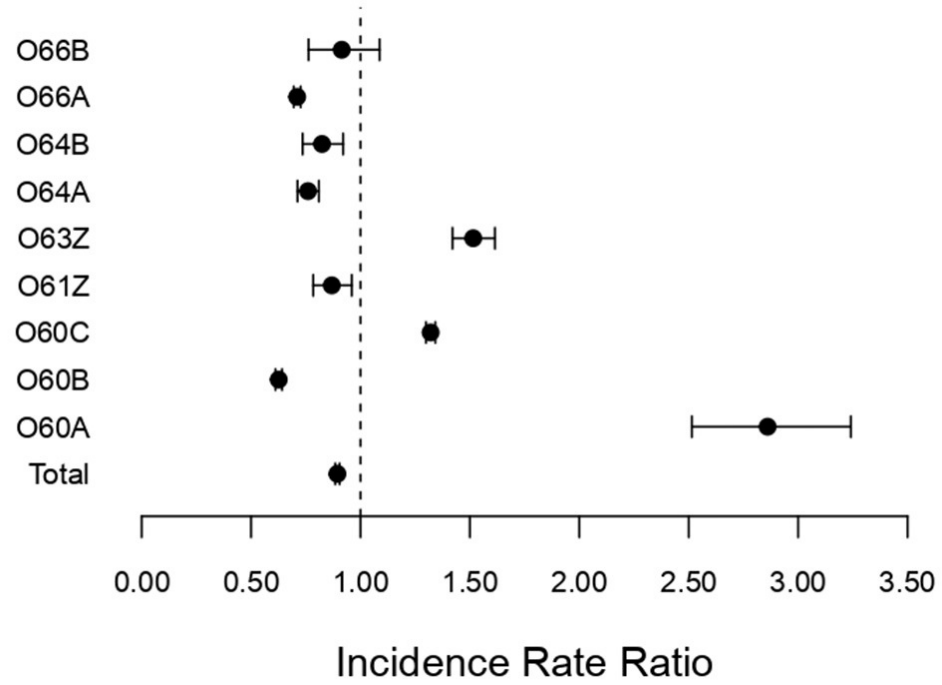
Incidence rate ratio (IRR) for non-surgical DRGs (O60A–O66B) associated with pregnancy, childbirth, and the puerperium during the pandemic (2020–2022) compared to pre-pandemic (2017–2019); incidence rate ratio with 95% confidence limits.

Figure 2 shows the corresponding IRRs calculated for non-surgical DRGs (O60A-O66B) associated with pregnancy, childbirth, and the puerperium.

During the pandemic, there were 13,430 patients treated surgically because of diseases and disorders of the female reproductive system. Compared to the pre-pandemic period, there is an average drop of 18.43% when the total number of patients was 16,465. During the pandemic, 7,886 (58.72%) patients were surgically treated at the tertiary health care level, and 5,545 (41.28%) at the secondary. The number of patients dropped by 16% at the tertiary and by 11% at the secondary health care level.

The decrease greater than average is related to N02A by 19% (*p* = 0.138546), N04Z by 19% (*p* < 0.0001), N03A by 31% (*p* = 0.034861), N07Z by 24% (*p* < 0.0001), N09Z by 26% (*p* < 0.0001), N10Z by 20% (*p* < 0.0001), N11B by 21% (*p* = 0.002066). An increase of 1% and 4% was observed in groups N02B and N01Z (*p* = 0.793676, *p* = 0.718742, respectively).

Table 3 compares the average number of total surgical DRGs (N01Z-N11B) during the pre-pandemic (2017–2019) and pandemic years (2020–2022) related to diseases and disorders of the female reproductive system.

**Table 3:** Comparison of surgical DRGs (N01Z-N11B) related to diseases and disorders of the female reproductive system done during pre-pandemic (2017–2019) and pandemic (2020–2022).

**Codes**	**2017–2019**		**2020–2022**		**2017–2019**	**2020–2022**	**Pre-pandemic–pandemic comparison**			***p*-value**
	**T**	**S**	**T**	**S**	**Average all hospitals**	**Average all hospitals**	**% Rate change T**	**% Rate change S**	**% Rate change all**	
N01Z	135	33	137	38	168	175	2%	13%	4%	0.718741542
N02A	69	42	58	32	111	90	−17%	−22%	−19%	0.138546415
N02B	527	299	517	320	826	837	−2%	7%	1%	0.793675662
N03A	44	35	26	28	79	54	−40%	−19%	−31%	0.034861147
N03B	536	393	556	479	929	1,035	4%	22%	11%	0.016432082
N04Z	1,255	1,058	1,019	855	2,313	1,874	−19%	−19%	−19%	1.1655E−11
N05A	9	6	9	4	15	13	−4%	−37%	−17%	0.614294665
N05B	625	385	548	317	1,011	865	−12%	−18%	−14%	0.000769804
N06Z	498	424	428	360	923	788	−14%	−15%	−15%	0.001130146
N07Z	3,949	1,941	2,733	1,727	5,891	4,460	−31%	−11%	−24%	6.48083E−45
N08Z	177	72	163	51	249	214	−8%	−30%	−14%	0.103824642
N09Z	1526	882	1028	754	2409	1782	−33%	−15%	−26%	3.85451E−22
N10Z	392	429	288	367	821	655	−26%	−14%	−20%	1.62045E−05
N11A	266	87	198	101	353	298	−26%	16%	−15%	0.032192897
N11B	223	146	178	111	368	289	−20%	−24%	−21%	0.002066388

Codes: N01Z–N11B refers to surgical DRGs related to diseases and disorders of the female reproductive systems; A cases with catastrophic complications and comorbidities, B cases without catastrophic or severe complications and comorbidities, Z-no comorbidities or complications taken into account, procedural DRG group.

T, tertiary hospitals (University Clinical Centers, Clinical hospitals); S, secondary hospitals (General and County hospitals).

**Figure 3:**
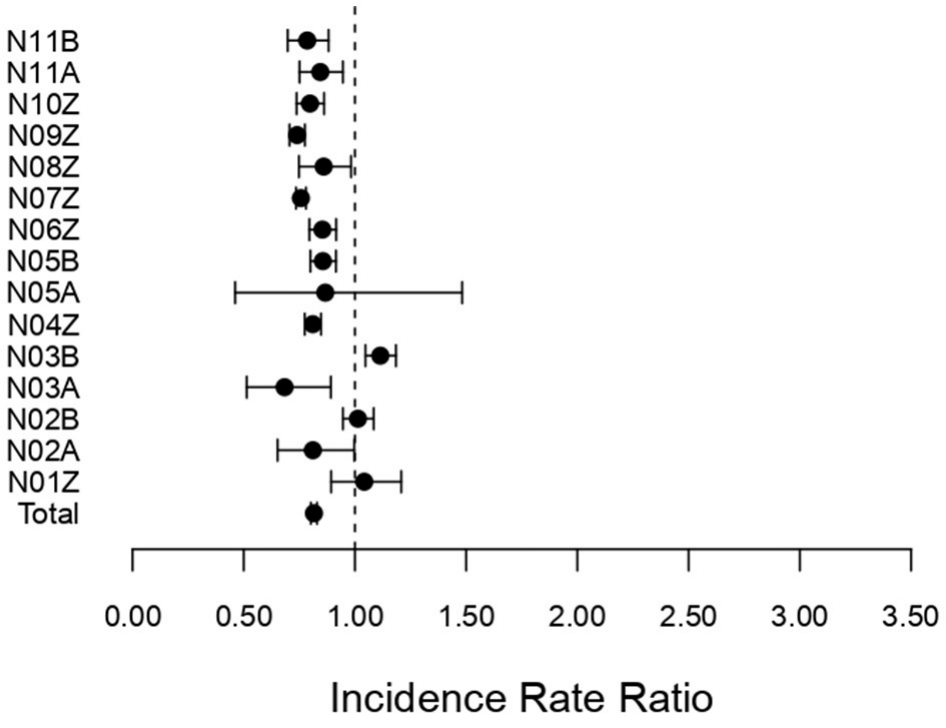
Incidence rate ratio (IRR) for surgical DRGs (N01Z–N11Z) associated with diseases and disorders of the female reproductive system during the pandemic (2020–2022) compared to pre-pandemic (2017–2019). Incidence rate ratio with 95% confidence limits.

Figure 3 shows the corresponding IRRs calculated for surgically treated diseases and disorders of the female reproductive system.

During the pandemic, there were 2,523 patients treated non-surgically because of diseases and disorders of the female reproductive system. Compared to the pre-pandemic period, there is an average drop of 35.06% when the total number of patients was 3,885. During the pandemic, 1,433 (56.8%) patients were non-surgically treated at the tertiary healthcare level, compared to 1,090 (43.2%) patients at the secondary. The number of patients dropped by 35% at the tertiary and by 21% at the secondary health care level.

A decrease greater than average is observed in groups N62A by 41% (*p* = 0.006157) and N62B by 40% (*p* < 0.0001). The decrease lower than average is noticed in groups N60A by 14 % (*p* = 0.235858), N60B by 34% (*p* < 0.0001), and N61Z by 21 % (*p* = 0.015565).

Table 4 compares the average number of total non-surgical admissions during the pre-pandemic (2017–2019) and pandemic years (2020–2022) related to diseases and disorders of the female reproductive system.

**Table 4:** Comparison of non-surgical DRGs (N60A-N62B) related to diseases and disorders of the female reproductive system done during pre-pandemic (2017–2019) and pandemic (2020–2022).

**Codes**	**2017–2019**		**2020–2022**		**2017–2019**	**2020–2022**	**Pre-pandemic–pandemic comparison**			***p*-value**
	**T**	**S**	**T**	**S**	**Average all hospitals**	**Average all hospitals**	**% Rate change T**	**% Rate change S**	**% Rate change all**	
N60A	86	57	65	58	143	123	−24%	3%	−14%	0.235858256
N60B	1,425	486	826	430	1,911	1,255	−42%	−12%	−34%	2.23832E−31
N61Z	100	130	61	119	230	181	−39%	−8%	−21%	0.015565191
N62A	32	40	21	21	72	43	−34%	−46%	−41%	0.006156635
N62B	714	816	459	461	1,530	921	−36%	−43%	−40%	8.7465E−35

Codes: N60A–N62B refers to no surgical DRGs related to diseases and disorders of the female reproductive systems; A cases with catastrophic complications and comorbidities, B cases without catastrophic or severe complications and comorbidities, Z-no comorbidities or complications taken into account, procedural DRG group.

T, tertiary hospitals (University Clinical Centers, Clinical hospitals); S, secondary hospitals (General and County hospitals).

**Figure 4:**
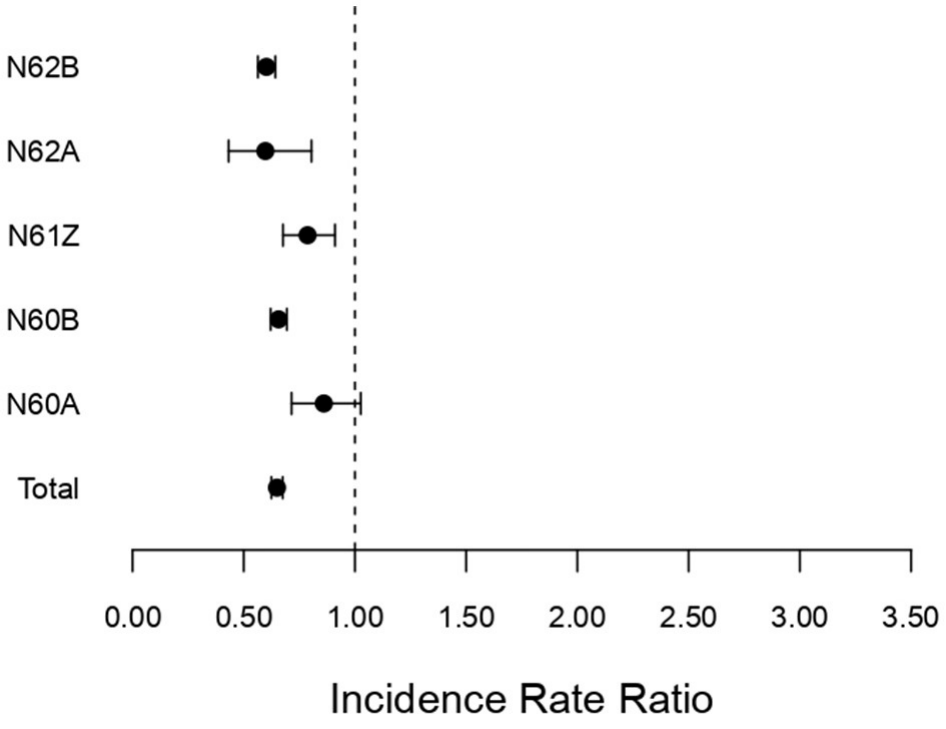
Incidence rate ratio (IRR) for non-surgical (N60A–N62B) associated with diseases and disorders of the female reproductive system during the pandemic (2020–2022) compared to pre-pandemic (2017–2019). Incidence rate ratio with 95% confidence limits.

Figure 4 shows the corresponding IRRs calculated for non-surgically treated diseases and disorders of the female reproductive system.

## Discussion

During the pandemic period in Croatia, a significant average decrease was noted across acute cases classified by DRGs. Specifically, there was a 6.64% decrease in surgically treated patients with conditions related to pregnancy, childbirth, and the puerperium, and there was a 10.56% decrease in non-surgically treated patients within the same categories.

DRG data also showed that the COVID-19 period also witnessed a significant decrease in cases related to disorders of the female reproductive system. The decrease was 18.43% for surgically treated patients and 35.06% for non-surgically treated patients.

The first COVID-19 case in Croatia was confirmed on February 25, 2020. Three weeks later, hospital care delivery was reorganized to address the growing demands of the pandemic following a significant increase in COVID-19 cases.

In Zagreb, the capital of Croatia, three hospitals were designated as COVID-19 centers, and patients with illnesses associated with the COVID-19 who require inpatient care have been admitted there. The majority of hospitals established COVID-19 isolation units, and four comparable facilities have been established around the region (24).

In addition to the reorganization of the healthcare delivery system, tougher lockdown rules were applied in 2020. Hospitals' lower priority for elective treatments, a fall in the non-emergency admission referral rate due to fewer outpatient hours, and a shortage of hospital staff are all contributing causes. Another concern is the possibility of contracting COVID-19 in a hospital environment (25).

Furthermore, Zagreb suffered a further catastrophe in March 2020: a catastrophic earthquake that devastated some hospitals and gynecology departments.

By comparison internationally, Carbone et al. (26). in Italy conducted a review and meta-analysis and they found that during the lockdown periods, the pooled proportion of hospitalizations for any obstetrical or gynecological condition increased from 22.7 to 30.6%, with delivery increasing from 48.0 to 53.9%. In particular, they discovered that the pooled proportion of pregnant women suffering from hypertensive disorders rose (2.6% vs. 1.2%), as did women experiencing imminent labor (52% vs. 43%) and early rupture of membranes (12.0% vs. 9.1%). In contrast, they discovered reductions in the proportion of women with pelvic discomfort (12.4% vs. 14.4%), suspected ectopic pregnancy (1.8% vs. 2.0%), diminished fetal movements (3.0% vs. 3.3%), and vaginal bleeding for both obstetrical (11.7% vs. 12.8%) and gynecological (7.4% vs. 9.2%) issues (26).

Another study, similar in design, examined the characteristics and outcomes of patients undergoing elective laparoscopic cholecystectomy for benign gallbladder disease in a single secondary care hospital in the UK before the COVID-19 pandemic and during the recovery phase of the pandemic (27). The authors also observed a significant delay in elective procedures due to fear of COVID-19 and hospital reorganization. Thus, a significantly lower proportion of patients underwent total cholecystectomy during the recovery phase of the pandemic (*n* = 49; 92.5%) compared to patients who underwent surgery before the pandemic (*n* = 106; 99.1%; *p* = 0.04) (27). In addition, another retrospective epidemiologic cohort study of a single level I trauma center in northern Italy found that overall orthopedic surgical activity decreased by 72.4% during the lockdown period (from 36 ± 6.1 to 10.7 ± 8.4 per week; *p* < 0.01), with the ratio of emergency to elective surgery increasing from 0.7:1 in 2019 to 3.3:1 in 2020 (28). In addition, elective surgery was almost completely suspended and was affected with a decrease of 88.9%, while emergency trauma surgery suffered a decrease of 49.7%.

During the lockdown time at the tertiary hospital level in southern Italy, it was also noticed that, similar to the Croatian pattern, there was an overall decline in the number of obstetric and gynecologist emergency visits (29). Furthermore, pregnant women declined to undergo prenatal invasive diagnostic procedures, even though the number of births remained constant and even increased during the lockdown, demonstrating that women came to the hospital when they had no other choices (14).

Carbone et al. observed a rise in hospitalizations as well, particularly for pregnancy-related hypertension problems and labor signs. Given that contractions and vaginal discharge are among the most common reasons for seeking emergency care, the discovery of increased hospitalizations for these conditions during the lockdown could be interpreted as evidence of a reduction in the number of unnecessary visits for unclear conditions, which were the cause of emergency unit overload. As a result, it appeared that people only sought medical assistance when they had true, specific labor signs and a genuine need. The studies that examined this topic found an increase in hypertensive disorders, and although this hasn't been proven, it may be due to the more sedentary lifestyle that was forced upon people during the lockdown, as well as the eventual reduction in antenatal care appointments, which resulted in missed antenatal screenings (26).

During the COVID-19 pandemic, a significant decrease in gynecologic procedures and ambulatory obstetrical and gynecologic visits at two big health systems in United States of America was observed. The decrease in surgical volume was notably noticeable from April 5 to June 27, 2020. During this time, the most stringent institutional and regional regulations were in place, preventing or severely limiting elective surgery. Despite a rise in surgery volume in the second part of the year, surgical caseload for 2020 did not return to pre-pandemic levels. Similarly, early in the pandemic, ambulatory care decreased significantly, and quantities of various forms of ambulatory care did not return to 2019 levels (30). During this public health crisis, gynecologic surgical techniques were most likely impacted by published professional society guidelines for changing surgical practice during the COVID-19 pandemic (31, 32).

Concerns have been raised in cancer, gynecologic, and obstetric literature concerning the possible unfavorable health effects linked with deferred or delayed care. Deferred or delayed treatment has been linked to more advanced breast and cervical cancers at the time of diagnosis (33). The impact of COVID-19 on breast and colon cancer screening and diagnosis over the next decade predict a 1% increase in cancer mortality (34). Furthermore, while sexual distance may initially result in a reduction in diagnosis of sexually transmitted illnesses during the pandemic, restricted access to testing and treatment is predicted to result in a subsequent rise in the rates of diagnosed sexually transmitted infections (35).

In the context of the COVID-19 outbreak, rapidly growing modern technologies have brought with them tremendous potential for many applications that will change the way we work, learn and live globally. Among them, healthcare is one such specific industry that is undergoing an interesting transformation with the integration of these telecommunication technologies. Several publications have hinted at the development and implementation of different variants of telemedicine during the COVID-19 outbreak, with the aim of offsetting the decline in hospital visits, especially for non-surgical cases (36–39). The most important examples are: telemedicine in emergency cases and triage, telemedicine in critical care, telemedicine-assisted follow-up and rehabilitation, telemedicine in palliative care and general telemedicine for elderly (36–39). Different modalities and use of telehealth technologies in the prenatal and postpartum periods during COVID-19 pandemic undoubtedly proved that integration of telehealth into maternity care is needed. The potential to reduce disparities in care and clinical outcomes should be further harnessed by step wise implementation based on the well designed policy and stakeholders engagement.

## Strengths and limitations

The use of a full data set on inpatient activity for all gynecology departments in Croatia is the study's principal strength. We also discussed how the SARS-CoV-2 pandemic directly affected the number of patients with gynecological illnesses who were admitted to hospitals, as well as the number of patients who had surgery and received non-surgical care at the secondary and tertiary levels of care. However, there are some limitations to our analysis. DRG data lack detailed, granular information on potentially significant variables or confounders, such as patient age and co-morbidities. Instead, they offer large-scale, descriptive insights into the utilization of gynecology department services. Furthermore, DRG reporting in Croatia is mandatory only for hospitals contracted by the Croatian Health Insurance Fund. As a result, we were unable to include maternity data from one private hospital. However, this facility reports only ~500 inpatient maternity cases annually, and their exclusion is unlikely to significantly impact the reported results.

Finally, the study design employs a cross-sectional design, which captures data at specific points in time or aggregated over fixed periods. While this approach allows for the analysis of associations between variables (e.g. the impact of COVID-19 pandemic on gynecological and obstetric admissions), it is inherently limited in its ability to establish definitive causal relationships. The primary limitation of cross sectional studies in this regard is their inability to track temporal sequences. To improve causal inference, future research could consider longitudinal cohort studies but aggregated, publicly available DRG data can not be used for a such purpose.

## Conclusion

This is the first research that, to our knowledge, demonstrates how the COVID-19 pandemic has affected inpatient treatment for patients in Croatia who have gynecological issues associated to pregnancy, delivery, and the puerperium as well as illnesses and abnormalities of the female reproductive system. At secondary and tertiary hospital levels, we noticed a statistically significant average decline in the overall number of admissions as well as the number of gynecological patients who were admitted to the hospital. In future pandemic scenarios, it will be important for obstetrics and gynecology practitioners to develop innovative strategies to maintain or improve patient access to care while ensuring stringent safety measures to prevent infection transmission among patients and healthcare personnel.

## Data Availability

The raw data supporting the conclusions of this article will be made available by the authors, without undue reservation.

## Footnotes

^1^MDC 13 AR-DRG groups related to diseases and disorders of female reproductive system: N01Z Pelvic Evisceration and Radical Vulvectomy; N02AUterine, Adnexa Procedure for Ovarian or Adnexal Malignancy W CC N02B Uterine, Adnexa Procedure for Ovarian or Adnexal Malignancy W/O CC N03A Uterine, Adnexa Procedure for Non-Ovarian or Adnexal Malignancy W CC N03B Uterine, Adnexa Procedure for Non-Ovarian or Adnexal Malignancy W/O CC N04ZHysterectomy for Non-Malignancy N05A Oophorectomies and Complex Fallopian Tube Procs for Non-Malig W Cat or Sev CC N05B Oophorectomies and Complex Fallopian Tube Procs for Non-Malig W/O Cat or Sev CCN06Z Female Reproductive System Reconstructive ProceduresN07Z Other Uterine and Adnexa Procedures for Non-Malignancy N08ZEndoscopic and Laparoscopic Procedures for Female Reproductive System N09Z Conisation, Vagina, Cervix and Vulva Procedures N10ZDiagnostic Curettage or Diagnostic Hysteroscopy N11A Other Female Reproductive System OR Procs Age >64 or W Malignancy or W CC N11B Other Female Reproductive System OR Procs Age < 65 W/O Malignancy W/O CC N60AMalignancy, Female Reproductive System W Catastrophic or Severe CC N60BMalignancy, Female Reproductive System W/O Catastrophic or Severe CC N61ZInfections, Female Reproductive System N62AMenstrual and Other Female Reproductive System Disorders W CC N62B Menstrual and Other Female Reproductive System Disorders W/O CC.

^2^MDC 14 AR-DRG groups related to pregnancy, childbirth and puerperium O01ACaesarean Delivery W Catastrophic CC O01BCaesarean Delivery W Severe CC O01CCaesarean Deliver W/ Catastrophic or Severe CC O02AVaginal Delivery W OR Procedure W Catastrophic or Severe CC O02BVaginal Delivery W OR Procedure W/O Catastrophic or Severe CC O03ZEctopic Pregnancy O04Z Postpartum and Post Abortion W OR Procedure O05Z Abortion W OR Procedure O60AVaginal Delivery W Catastrophic or Severe CC O60B Vaginal Delivery W/O Catastrophic or Severe CC O60C Vaginal Delivery Single Uncomplicated W/O Other Condition O61Z Postpartum and Post Abortion W/O OR Procedure O63Z Abortion W/O OR Procedure O64A False Labour Before 37 Weeks or W Catastrophic CC O64B False Labour After 37 Weeks W/O Catastrophic CC O66AAntenatal and Other Obstetric Admission O66B Antenatal and Other Obstetric Admission, Sameday.
